# The Enrichment of Whey Protein Isolate Hydrogels with Poly-γ-Glutamic Acid Promotes the Proliferation and Osteogenic Differentiation of Preosteoblasts

**DOI:** 10.3390/gels10010018

**Published:** 2023-12-23

**Authors:** Daniel K. Baines, Varvara Platania, Nikoleta N. Tavernaraki, Mattia Parati, Karen Wright, Iza Radecka, Maria Chatzinikolaidou, Timothy E. L. Douglas

**Affiliations:** 1 Faculty of Science and Technology, School of Engineering, Lancaster University, Gillow Avenue, Lancaster LA1 4YW, UK; d.baines3@lancaster.ac.uk; 2 Faculty of Health and medicine, Division of Biomedical and Life Sciences, Lancaster University, Gillow Avenue, Lancaster LA1 4YW, UK; karen.wright@lancaster.ac.uk; 3 Department of Materials Science and Technology, University of Crete, GR-70013 Heraklion, Greece; plataniavarvara@yahoo.com (V.P.); nikoleta.natalia@gmail.com (N.N.T.); mchatzin@materials.uoc.gr (M.C.); 4 Faculty of Science and Engineering, School of Life Sciences, University of Wolverhampton, Wolverhampton WV1 1LY, UK; m.parati@wlv.ac.uk (M.P.); i.radecka@wlv.ac.uk (I.R.); 5 Institute of Electronic Structure and Laser, Foundation for Research and Technology Hellas, GR-70013 Heraklion, Greece

**Keywords:** whey protein, γ-PGA, bone scaffolds, Raman, swelling, biocompatibility, ALP, collagen, osteogenesis, osteogenic differentiation, bone tissue engineering

## Abstract

Osseous disease accounts for over half of chronic pathologies, but there is a limited supply of autografts, the gold standard; hence, there is a demand for new synthetic biomaterials. Herein, we present the use of a promising, new dairy-derived biomaterial: whey protein isolate (WPI) in the form of hydrogels, modified with the addition of different concentrations of the biotechnologically produced protein-like polymeric substance poly-γ-glutamic acid (γ-PGA) as a potential scaffold for tissue regeneration. Raman spectroscopic analysis demonstrated the successful creation of WPI-γ-PGA hydrogels. A cytotoxicity assessment using preosteoblastic cells demonstrated that the hydrogels were noncytotoxic and supported cell proliferation from day 3 to 14. All γ-PGA-containing scaffold compositions strongly promoted cell attachment and the formation of dense interconnected cell layers. Cell viability was significantly increased on γ-PGA-containing scaffolds on day 14 compared to WPI control scaffolds. Significantly, the cells showed markers of osteogenic differentiation; they synthesised increasing amounts of collagen over time, and cells showed significantly enhanced alkaline phosphatase activity at day 7 and higher levels of calcium for matrix mineralization at days 14 and 21 on the γ-PGA-containing scaffolds. These results demonstrated the potential of WPI-γ-PGA hydrogels as scaffolds for bone regeneration.

## 1. Introduction

Osseous-associated defects are one group of chronic diseases, accounting for half of the chronic pathologies in individuals over the age of 50 and affecting around 200 million people worldwide [1]. They are normally the result of fractures, a consequence of weakened bones caused by age-related osteoporosis [2]. Although bone possesses regenerative qualities, the possibilities are limited. Larger defects result in impaired healing and the failure to regenerate significant gaps in the bone [3]. Bone autografts are the gold standard treatment [4]; however, the use of autografts presents limitations including a limited availability, donor site morbidity, and long operation times [5]. Additionally, immune rejection and non-union present further complications [6]. Therefore, there is a requirement for scaffold-forming materials allowing for bone regeneration, resulting in the emergence of tissue engineering as a promising alternative to autologous bone grafting [7]. Potential polymers to be utilised as osteogenic scaffolds, whether natural or synthetic polymers, require certain properties. Ideally, any scaffold should share properties similar in composition, structure, and functionality to the extracellular matrix (ECM) [8]. The polymers should be bioactive, compatible, degradable, load bearing, osteoconductive, and have the potential for localised drug delivery [9]. Numerous natural or synthetic polymers have been developed including hybrid materials. However, many fail to meet all requirements for use in bone tissue engineering. They fail to produce satisfactory mechanical properties or fail to be biologically active [10,11]. Nevertheless, a promising new biomaterial, whey protein isolate (WPI), has proven to be a potential candidate for osteo support and regeneration.

**Figure 1:**
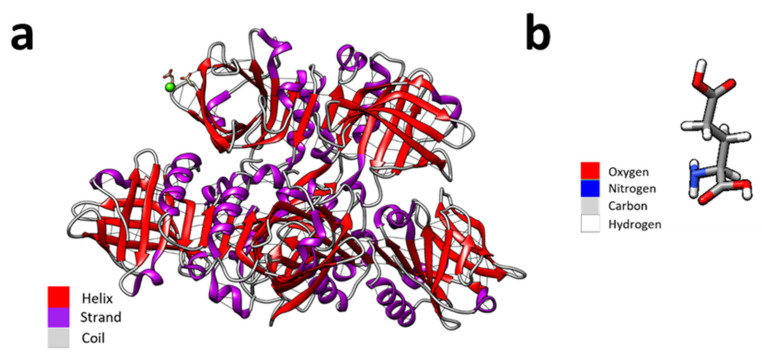
(**a**) Coloured depiction of the secondary structures of beta-lactoglobulin. Here, the helix is depicted as red, the strands in purple, and coils in grey. A calcium ion (green) is depicted interacting with a β-lactoglobulin receptor. (**b**) A glutamic acid monomer coloured by element, with oxygen in red, nitrogen in blue, carbon in grey, and hydrogen in white. Molecular graphics and analyses were performed with UCSF Chimera [19]. The beta-lactoglobulin molecular structure was sourced from PubMed and the glutamic acid molecule was sourced from PubChem.

Formally, WPI is derived from a waste product in the dairy industry and contains purified proteins of whey with the main component being β-lactoglobulin, which is shown in Figure 1 [12]. The main potential of WPI as a biomaterial comes from the ability of WPI to produce pliable and sterilisable hydrogels through heat or pressure induction [13,14]. One of the advantages of a hydrogel is the ease of incorporation of water-soluble molecules in the water phase. Although WPI does support cell attachment and proliferation, most studies have involved the formation of hybrid composites with the hydrogels. For instance, in [15], cytocompatible WPI-bioactive glass composites were produced, supporting MG-63 osteoblast cellular functioning. In [16], WPI–WPI Urease hydrogels were synthesized, demonstrating that WPI-PG hydrogels support the growth of osteosarcoma-derived MC3T3-E1 cells. Hence, such WPI-PG hydrogels may provide a medicinal route to restrict microbial infections, whilst presenting desirable mechanical properties and promoting stem-cell attachment. Similarly, osteogenic behaviour was observed in [17], in which WPI–hydroxyapatite hydrogels were synthesized as a potential scaffold for bone substitution. Furthermore, WPI–aragonite composites produced ECM-like mineralisation and enabled MG-63 proliferation [18].

**Figure 2:**
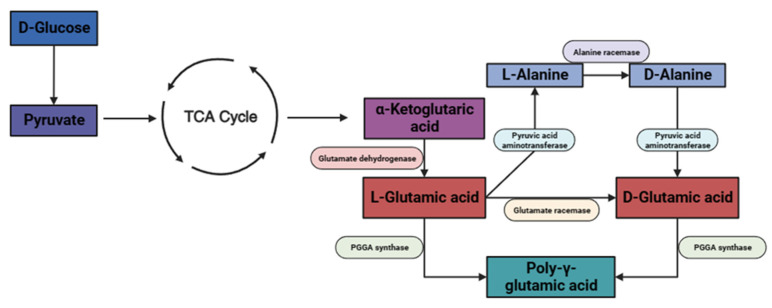
A schematic of the bacterial metabolic production of γ-PGA via the citric acid cycle (TCA). The image was created on Biorender.com.

Poly-γ-glutamic acid (γ-PGA) is a biopolymer produced primarily by bacterial Gram-positive bacteria, predominately those of the *Bacillus* species [20]. γ-PGA was first identified in a capsule of *Bacillus anthracis*. Subsequently, it was isolated from several other microbes such as *Bacillus licheniformis*, *Bacillus subtilis natto*, *Rhodopirellula baltica*, as well as *Staphylococcus epidermidis*. The mechanism of bacterial synthesis can be observed in Figure 2. The polymer consists of multiple repeating L-glutamic acid and D-glutamic acid amino acid monomers [21]. The hydrophilic polymer has multiple interesting qualities including immunogenicity, nontoxicity, and biodegradability [22]. Previously, γ-PGA has demonstrated functions compatible with osteoblastic cellular proliferation. For instance, γ-PGA combined with bioactive glass was found to have a supporting role for SaOs-2 osteosarcoma cell proliferation [23]. Recently, Parati et al. [24] demonstrated a protective role of γ-PGA on tooth enamel by providing an inhibitory effect on calcium dissolution, significantly reducing the loss of hydroxyapatite.

It would be beneficial if a scaffold would prevent microbial infection. γ-PGA has demonstrated antimicrobial activity with Zu et al. [25], suggesting that γ-PGA demonstrates a minimum inhibitory concentration of <2.5mg/mL against Gram-positive and Gram-negative species of the bacteria *B. subtilis* and *Escherichia coli*, respectively. However, the present antimicrobial activity was molecular mass dependent [26]. Previously, Gamarra-Montes et al. [26] had demonstrated the antibacterial properties of γ-PGA against *S. aureus*, *L. monocytogenes*, *E. coli*, and *P. aeruginosa*, known infection-causative agents.

In this study, a novel approach was taken: WPI hydrogels were combined with γ-PGA to evaluate any potential for WPI–γ-PGA hydrogels to be utilised as scaffolds for bone regeneration. Raman spectroscopy was utilised to ascertain the incorporation of γ-PGA into the WPI. Swelling assays and mechanical testing were performed to determine whether the addition of γ-PGA influences the structural behaviour of the hydrogels. Additionally, a cytocompatibility assessment was performed to determine the adhesion, viability, and proliferation of preosteoblastic cells cultured onto hydrogels, and the expression of bone-related markers was evaluated to demonstrate the potential of the hydrogels to promote osteogenic differentiation.

## 2. Results and Discussion

### 2.1. Raman Spectroscopy

**Figure 3:**
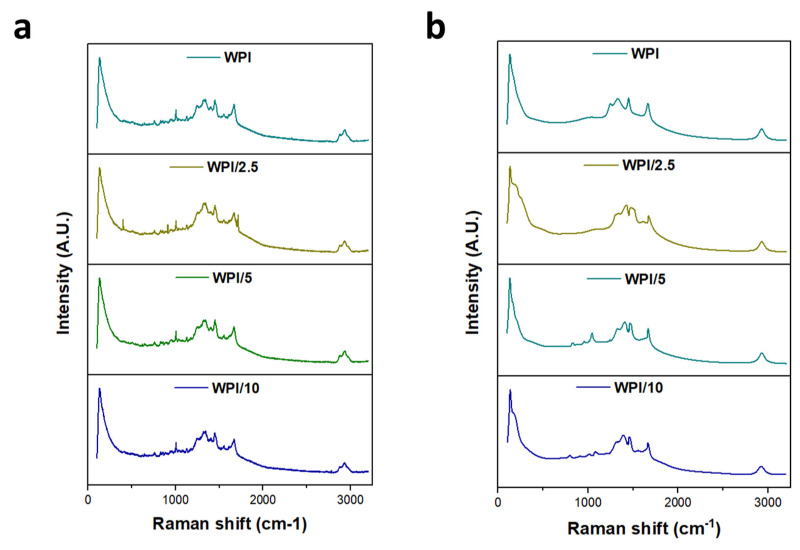
(**a**,**b**) Raman spectroscopy analysis pre- and post-normalisation, observable in ascending order and denoted by their γ-PGA concentration: WPI = 0%, WPI/2.5 = 2.5%, WPI/5 = 5%, and WPI/10 = 10% γ-PGA (n = 5).

**Figure 4:**
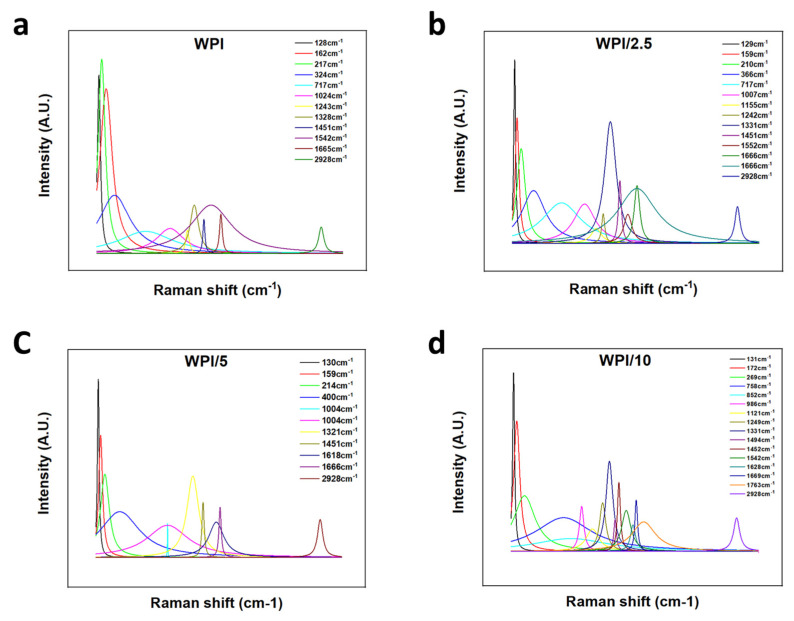
(**a**–**d**) Relevant peaks post-convergence. The peaks demonstrate the underlying molecular interactions which form the associated peaks displayed by the Raman spectroscopy results. Confidence in the results was demonstrated through Chi-squared and R-squared values: (**a**)—0.99619 (CHI^2^ 1.16768 × 10^−4^), (**b**)—0.98978 (CHI^2^ 3.64211 × 10^−4^), (**c**)—0.9937 (CHI^2^ 1.94365 × 10^−4^), and (**d**)—0.99387 (CHI^2^ 1.80616 × 10^−4^).

**Figure 5:**
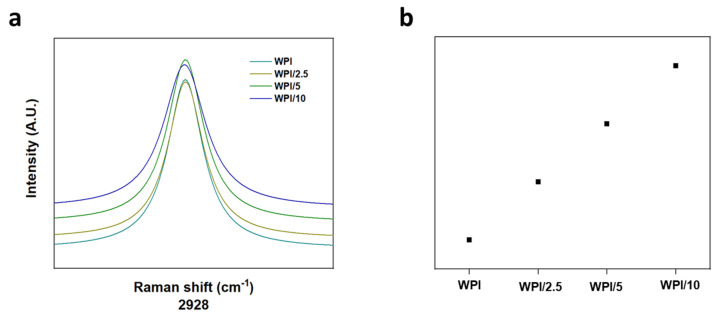
(**a**,**b**) An example of the peaks broadening with increasing γ-PGA concentrations at 2928 cm^−1^. The observables are the following WPI–γ-PGA concentration variables: WPI = 0% γ-PGA, WPI/2.5 = 2.5% γ-PGA, 5WPI/5 = 5% γ-PGA, and WPI/10 = 10% γ-PGA.

Raman spectroscopy was employed to ascertain the incorporation of glutamic acid polymers into the WPI hydrogel, the results of which can be observed in Figure 3, Figure 4 and Figure 5. A search in the literature returned several peaks directly associated with glutamic acid, whether that be the L- or D- isomers of the molecule. The peaks acquired from the literature formed the basis for the analysis of the results. Although WPI itself has glutamic acid present and thus, glutamic acid-associated peaks would be present, an increase in the glutamic acid-associated peaks represented a positive result. Additionally, R-squared values and a Lorentzian fitting were utilised to determine the statistical viability of the results. The results from the Lorentzian fitting are observable in Figure 3a,b. Additionally, the acquired peaks post-convergence can be observed in Figure 4a–d. 

The results yielded seven peaks that were consistent throughout all γ-PGA concentrations and four peaks that converged for only one or more of the γ-PGA concentration variables. Table 1 is representative of these results. 

One glutamic acid-associated peak consistent throughout all γ-PGA concentration variables was present at 2928 cm^−1^; this was representative of CH_2_ stretching vibrations. No Raman shift was observed between any of the WPI-γ-PGA concentration variables, and the results were consistent with peaks suggested by Freire et al. [27]. However, Freire et al. suggested two strong peaks at 2938 cm^−1^ and 2974 cm^−1^. Here, we attained one strong peak at 2928 cm^−1^, suggesting a potential red shift from the 2938 cm^−1^ peaks suggested in [27]. Additionally, in [27], the L-isomer of glutamic acid was analysed specifically. Considering that γ-PGA is a complex of both D- and L-isomers, there was potential for the results to include interactions or spectra from the D-isomer, contributing to the results with the additional interactions from the additional amino acid constituents in WPI. Furthermore, as highlighted by Figure 5a,b, a red shift was observed as expected, with an increase in the γ-PGA concentration as demonstrated by the broadening of the peaks with an increase in γ-PGA.

At the opposite end of the spectrum were peaks associated with lattice rocking vibrations, again consistent with the results from Barth et al. [28], which were ascertained to be located below 199 cm^−1^. However, a blue shift was observed with each increasing concentration variable increase of 1 cm^−1^, respectively, to the closest lesser concentration. For instance, the WPI-0 γ-PGA variable returned results of 128 cm^−1^, WPI-2.5% γ-PGA was 129 cm^−1^, WPI-5% γ-PGA was 130 cm^−1^ and the WPI-10% variable was 131 cm^−1^ respectively. Similarly, Barth et al. [28] and Guangyong et al. [29] suggested peaks at 1319 cm^−1^, 1327 cm^−1^, 1346 cm^−1^, and 1379 cm^−1^; similar peaks were observable in the results with peaks at 1328 cm^−1^ for the 0 γ-PGA, 1338 cm^−1^ for the 2.5% γ-PGA variable, 1321 cm^−1^ for the 5% γ-PGA variable, and 1331cm^−1^ for the 10% variable. However, the influence for these peaks was associated with more than one potential molecular interaction. For instance, interactions in this region have been associated with COOH in plane bending vibrations, CH_3_ wagging vibrations, CH in plane bending vibrations, and COO– symmetric stretching vibrations. Potential discrepancies in the comparisons with [28,29] could be attributed to differing glutamic acid states utilised in the investigations, i.e., whether the glutamic acid is in a solid form, a solution, or its pure crystal form. Furthermore, coupled with amino acid interactions, the constituent WPI amino acids likely attributed to the shift demonstrated in the data presented. Therefore, the results demonstrate the successful incorporation of γ-PGA into the WPI to form viable WPI-γ-PGA hydrogels at the various concentrations. 

### 2.2. Swelling Analysis

**Figure 6:**
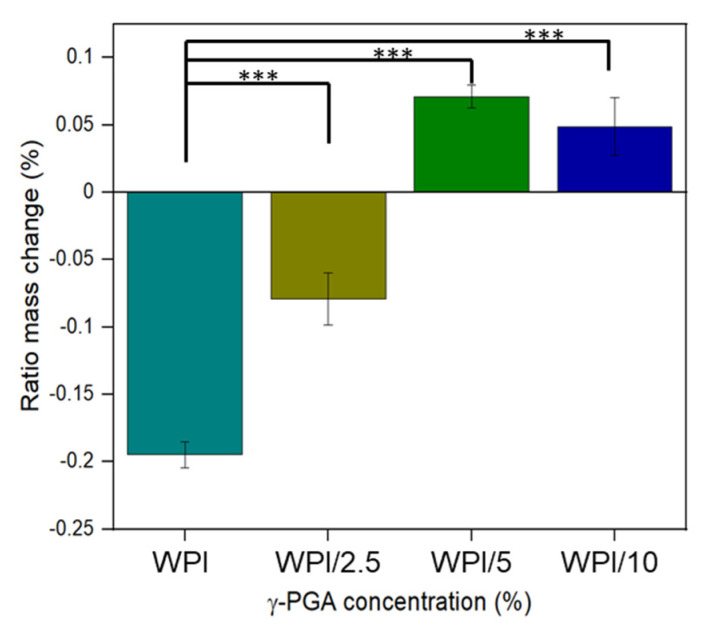
Polymer swelling assay under pH 7 conditions. The hydrogel samples were introduced to 5 mL of PBS solution. Each bar represents the mean ± SD of n = 10 (*** *p* < 0.001; compared to the WPI control). The observables are γ-PGA concentrations: WPI = 0%, WPI/2.5 = 2.5%, WPI/5 = 5%, and WPI/10 = 10% γ-PGA.

The results for the swelling assay are shown in Figure 6. The results indicate that the addition of γ-PGA influences the WPI hydrogels in a positive manner, as demonstrated by the increase in the amount of solution the hydrogels can uptake before degrading. However, the results demonstrated that the positive influence of the addition of γ-PGA is concentration dependent. The swelling potential increases from a mass percentage loss of −19.5% for the 0% γ-PGA control to a mass percentage of −7.9% for the 2.5% γ-PGA (*p* < 0.05). However, with the 2.5% γ-PGA, the hydrogels increased in mass rather than losing mass with the 5% γ-PGA samples, gaining a percentage increase of 7.1. However, the 10% γ-PGA sample displayed less swelling potential than the 5% γ-PGA sample, producing a swelling ratio percentage of 4.9% compared to 7.10% (*p* < 0.05).

Due to the novelty of the work featured in this manuscript, there is a lack of literature concerning the effect of the addition of γ-PGA to WPI hydrogels. However, given the hydrophilic nature of the γ-PGA molecule, it was expected that the addition of γ-PGA at lower concentrations would positively influence the swelling capacity of the hydrogels, and this is generally observed, with the addition of 2.5% and 5% γ-PGA improving the swelling potential of the hydrogels. WPI hydrogels were formed by heat-induced gelation due to the initial denaturing of the beta-lactoglobulin protein exposing the hydrophobic residues and sulphur-containing cystine and methionine residues. This results in hydrophobic interactions and disulphide bridges between the protein molecules and hydrogel formation. Unlike denatured beta-lactoglobulin, γ-PGA does not present hydrophobic regions or sulphur-containing groups. Hence, one can speculate that elevating the γ-PGA concentration beyond a certain concentration would impede protein–protein interactions and binding, and thus have a negative effect on the structural integrity of the hydrogels. In turn, this would lead to increased degradation and a decrease in mass. This may explain the loss observed in WPI/10; however, such a discussion remains speculative.

### 2.3. Compression Analysis

**Figure 7:**
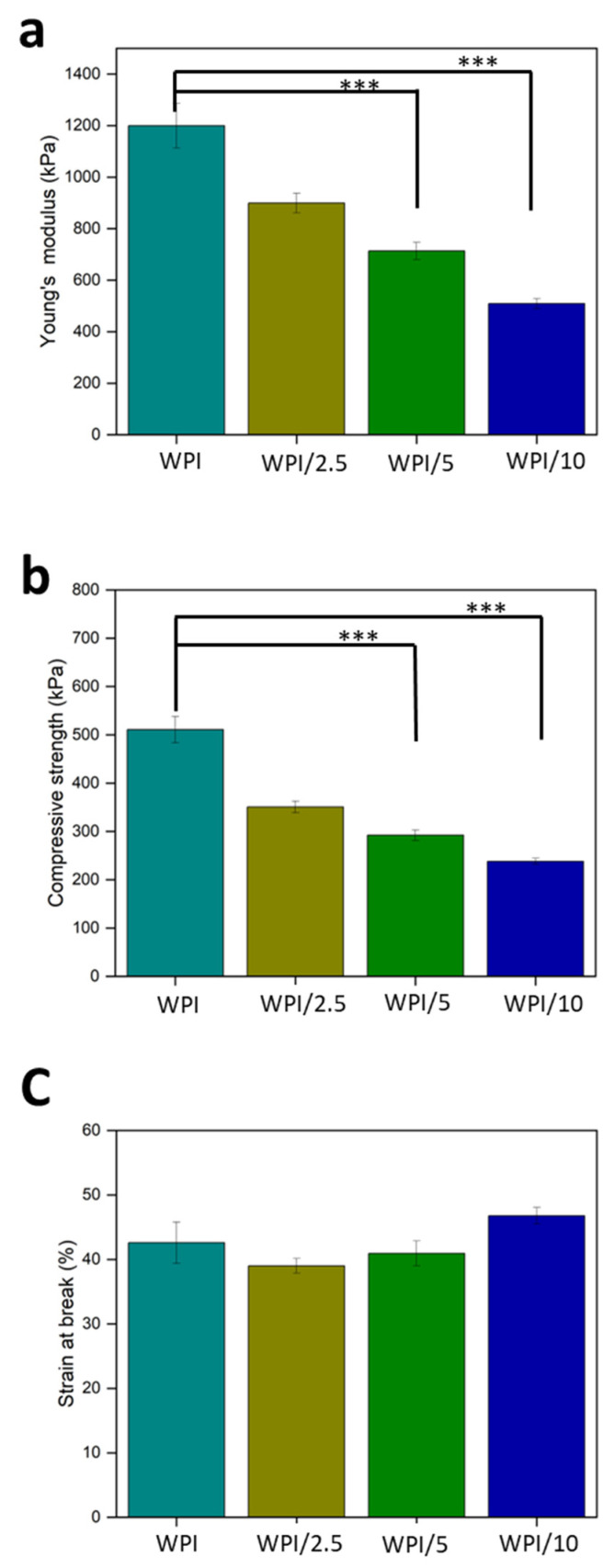
(**a**–**c**) The results of WPI-γ-PGA hydrogel compression testing. (**a**) Young’s modulus, (**b**) compressive strength, and (**c**) % strain at break. Each bar represents the mean ± SD of n = 10 (*** *p* < 0.001; compared to the WPI control). The observables are γ-PGA concentrations: WPI = 0%, WPI/2.5 = 2.5%, WPI/5 = 5%, and WPI/10 = 10%.

Load-bearing potential is a desired property of bone regenerative scaffolds. Therefore, a compression analysis was undertaken to ascertain the influence of the incorporation of γ-PGA on the mechanical strength of WPI hydrogels. Statistically significant differences were observed between the sample groups WPI/5 and WPI/10 (*p* < 0.05). However, the incorporation of γ-PGA into WPI hydrogels impacted the mechanical strength of the WPI hydrogels negatively. The results in Figure 7a display a clear concentration-dependent decrease in the load-bearing potential of the hydrogels, with an increase in the γ-PGA concentration leading to a decrease in the strength. Young’s modulus decreased from 1200 kPa for the WPI control group to 509 kPa for the WPI/10 sample group, resulting in a 58% loss in the structural strength (*p* < 0.05). This result is further supported by the lineal decrease in compressive strength in Figure 7b (*p* < 0.05). The concentration dependence is likely the result of the addition of nonhydrogel-forming amino acids diluting the potential for hydrophobic interactions or the formation of disulphide bridges, and thus weakening the structural integrity of the hydrogels. Additionally, the WPI hydrogel control group displayed values supported by previous work in the literature. The work here produced similar results to those presented by Ivory-Cousins et al. [30] when comparing WPI control samples. The WPI hydrogel demonstrated a Young’s modulus of approximately1200 kPa. However, ref. [30] presented data suggesting an increase in Young’s modulus of approximately 1300 kPa.

### 2.4. Biocompatibility and Osteogenic Capacity of the Scaffolds

**Figure 8:**
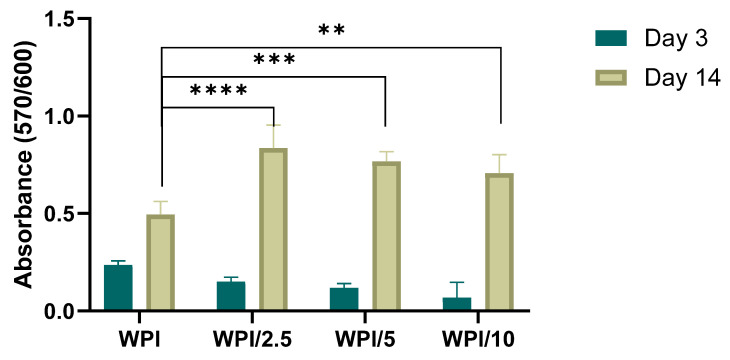
Preosteoblastic cell viability and proliferation on WPI and γ-PGA-containing hydrogels of various γ-PGA concentrations at days 3 and 14. Each bar represents the mean ± SD of n = 6 (** *p* < 0.01, *** *p* < 0.001, **** *p* < 0.0001; compared to the WPI control).

In vitro biocompatibility of the WPI and WPI-γ-PGA scaffolds was assessed using the PrestoBlue™ cell viability assay from day 3 to day 14, as shown in Figure 8. At day 3, a decrease in cell viability of the γ-PGA-containing scaffolds compared to the WPI control was observed; however, this was not significant. On day 14, a significant increase in the proliferation was observed for all three γ-PGA-containing scaffolds compared to the WPI control. Previous studies have recognized the potential of WPI for applications in tissue engineering, primarily regarding its cytocompatibility [31,32]. It has been previously reported that the incorporation of γ-PGA as a modifier not only enhanced the structural integrity of a polymer network, promoting material stability, but also fostered hydrophilicity, creating an environment conducive to cell attachment and proliferation [33,34]. Previous research on the dose response of 0.5–0.7 *w*/*v*% γ-PGA-modified glycerol hydrogels revealed significant enhanced cell proliferation and adhesion compared to the control hydrogels [34]. Another study reported on the significant increase of MC3TE-E1 cell proliferation on γ-PGA scaffolds containing 5 to 20 wt%, from day 1 to day 5 [35].

**Figure 9:**
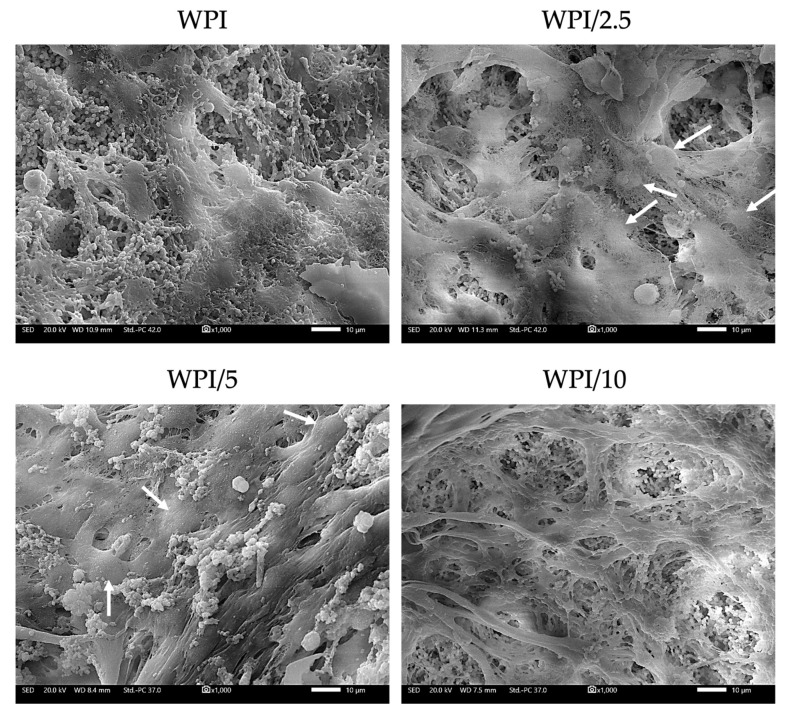
Visualization of preosteoblastic cell adhesion and morphology onto WPI-γ-PGA scaffolds with various γ-PGA concentrations on day 7. White arrows point to some visible cell nuclei. Scale bars represent 10 μM; magnification is ×1000.

The preosteoblastic cell adhesion and morphology were evaluated by means of scanning electron microscopy (SEM) imaging after 7 days in culture at ×1000 magnification (Figure 9). The preosteoblasts cultured on all three γ-PGA-containing scaffold compositions exhibited a strong attachment. On day 7, the formation of dense layers of cells with their characteristic elongated morphology was observed as were cell–cell interactions, expected to promote tissue formation. The cell nuclei are visible in the SEM images of WPI/2.5 and WPI/5, indicating the cell proliferative potential on the scaffolds. All three γ-PGA-containing scaffolds displayed a stronger cell attachment compared to the WPI control. These results are in line with previous studies investigating scaffolds containing γ-PGA, demonstrating that an increase in γ-PGA content from 0 to 20% *w*/*v* promotes cell adhesion [36].

**Figure 10:**
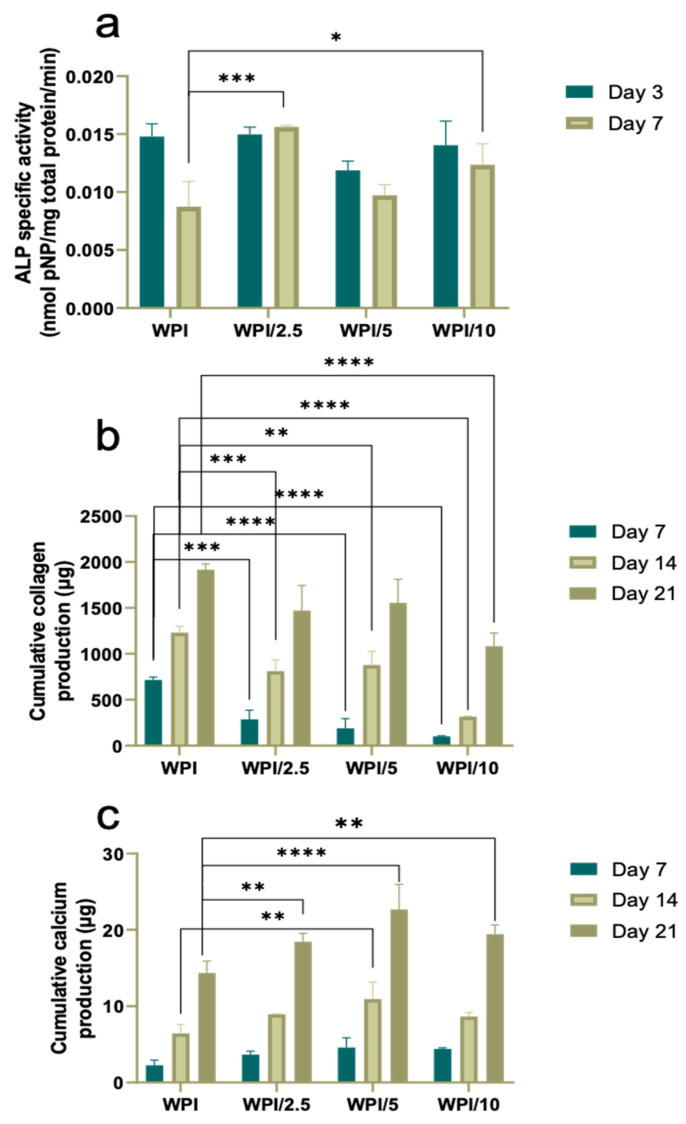
Assessment of the osteogenic potential of preosteoblastic cells cultured on γ-PGA-containing hydrogels over a period of 21 days. Expression of normalized ALP specific activity (**a**), collagen production (**b**), and calcium production (**c**) by preosteoblasts. Each bar represents the mean ± SD of n = 6 (* *p* < 0.05, ** *p* < 0.01, *** *p* < 0.001, **** *p* < 0.0001; compared to the WPI control).

Bone development is a process of the continuous deposition of calcium salts, accompanied by increased collagen mineralization. To evaluate WPI-γ-PGA scaffolds as prominent candidates for bone tissue engineering, a comprehensive in vitro study was conducted to assess the scaffolds’ osteogenic potentials. This involved the monitoring of alkaline phosphatase (ALP)-specific activity as an early marker of osteogenesis, calcium production as a late marker of osteogenesis, and quantification of the total secreted collagen, the main structural component of the ECM. The functionalization of WPI hydrogels with γ-PGA-induced ALP-specific activity at days 3 and 7 at all three concentrations, with a significant twofold enhancement for the WPI/2.5 sample group, and a 50% increase for the WPI/10 sample group are shown in Figure 10a. This result confirms the capacity of γ-PGA to promote the ALP activity of preosteoblasts, which is in line with previously reported [37] data on the use of high-molecular weight γ-PGA combined with bone morphogenetic protein 2, enabling its sustained release leading to the induction of ALP activity and other osteogenic differentiation markers. 

Total collagen levels in the supernatant of the cultured cells on the different WPI-γ-PGA hydrogels were quantified at different time points, as illustrated in Figure 10b. Collagen is a key structural component of the ECM, and deposition of a type I collagen-rich ECM is essential for the expression of specific osteoblast products, such as alkaline phosphatase, during the physiological developmental sequence of osteoblasts [38]. The enrichment of WPI hydrogels with γ-PGA resulted in a significant decrease in the measured collagen from the supernatants at days 7 and 14. γ-PGA is rich in carboxyl groups, while collagen possesses numerous amino, hydroxyl, and carboxyl groups. Ding et al. [39] suggested that that electrostatic and hydrogen bond interactions between γ-PGA and collagen molecules are paramount for the formation and stabilization of the γ-PGA/collagen bond. This may explain the decreased levels of collagen measured in supernatants of γ-PGA-containing scaffolds. Previously, Bu et al. [39] confirmed this hypothesis experimentally by introducing glutamic acid into collagen solutions, incubated at 37 °C. Their results suggested that increasing concentrations of glutamic acid from 50 to 200 mmol/L promoted collagen self-assembly and mineralization, resulting in the reduction of collagen levels in solution. Other reports also highlight the effect of glutamic acid on the collagen mineralization process by promoting collagen self-assembly [40]. Despite reduced collagen related to the presence of γ-PGA, the results in this study demonstrate that collagen production on all WPI–γ-PGA supernatants significantly increased at least fourfold from day 7 to day 21. This gradually increasing collagen secretion by cells suggests the active support of ECM formation.

Regarding calcium secretion, a significant enhancement has been observed in all three γ-PGA-containing scaffolds on day 21 compared to the WPI control shown in Figure 10c. The WPI/5 hydrogels showed the highest calcium values compared to all other scaffold compositions on day 14 and a significantly higher calcium production compared to the WPI control. From day 7 up to day 21, there was a gradual increase in the calcium concentration, indicating that preosteoblasts continued their differentiation into mature osteoblasts. Our results agree with other reports describing the excellent apatite-forming ability of γ-PGA due to the presence of carboxyl groups, which are effective for apatite nucleation [41,42]. Thus, the better deposition of calcium salts could be explained by the improved capture of calcium ions by the carboxylic groups of γ-PGA as part of the WPI–γ-PGA. The better osteogenic differentiation of MC3T3-E1 on the surface of WPI–γ-PGA scaffolds is also supported by the specific activity of ALP. All data consistently indicate that enrichment with γ-PGA enhances the differentiation of preosteoblasts into mature osteoblasts and expedites the biomineralization process. A similar improvement in biomineralization has been substantiated in various studies incorporating oligo/poly Glu. Notably, Karaman et al. [43] emphasized the positive impact of glutamic acid peptides bound to PLA/PLGA nanofibers on the nucleation of calcium phosphate and the osteogenic differentiation of bone marrow stromal cells. Averianov et al. [44] observed enhanced in vitro and in vivo biomineralization when nanocrystalline cellulose, modified with poly Glu, was employed as a filler for PLA or PCL.

## 3. Conclusions

The investigation has demonstrated the successful formation of WPI/γ-PGA hydrogels. Furthermore, through physiochemical and cellular analyses, the investigation provided evidence for the potential of WPI/γ-PGA hydrogels to be utilised as a novel material for bone regeneration.

Raman spectroscopy demonstrated the successful incorporation of γ-PGA into WPI hydrogels, as demonstrated by the observation of a concentration-dependent linearity in the broadening of the peak at 2928 cm^−1^. A physical characterisation of the hydrogels was performed; the swelling in PBS improved with an increasing γ-PGA concentration. The mechanical testing failed to demonstrate any improvement in the compressive strength. 

Based on the biological evaluation results, WPI/2.5 and WPI/5 indicated the highest cytocompatibility values and the formation of a dense cell layer. Particularly, WPI/2.5 displayed the highest ALP activity levels on day 7, and WPI/5 showed the highest calcium production on day 21. Although all three γ-PGA-containing scaffolds supported the biocompatibility and osteogenic differentiation of preosteoblasts, the two compositions WPI/2.5 and WPI/5 demonstrated the most promising results for bone tissue engineering applications. 

Therefore, the investigation suggests WPI/γ-PGA hydrogels as a primary candidate for further analysis for the purposes of osseous regenerative medicine.

## 4. Materials and Methods

### 4.1. Whey Protein Isolate–Poly-Gamma-Glutamic Acid Hydrogel Formation

**Figure 11:**
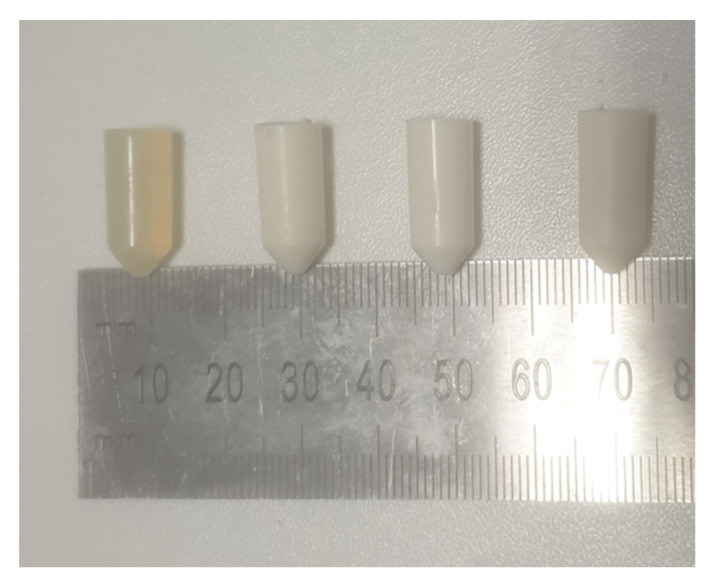
A depiction of WPI-γ-PGA hydrogels post sterilisation in ascending γ-PGA concentrations. From left to right: WPI, WPI/2.5, WPI/5, and WPI/10.

Whey protein isolate (WPI) sourced from Davis and CO. Foods international (Eden Prairie, MN, USA) and commercially available poly-gamma-glutamic acid, with a molecular mass of 440 kDa, were combined to fabricate hydrogels. The hydrogels were formed under heat-induced dissociation. The hydrogels were formed up to a concentration of 40% WPI (*w*/*v*) with MilliQ H_2_O. An additional 2.5%, 5%, or 10% γ-PGA was added to create the γ-PGA hydrogel variables, shown in Table 2. The acquired solutions were vortexed to begin homogenisation before being further homogenised utilising an IKA Loopster for 24 h. Gelation was then heat induced at 70 °C for 5 min for individual 1 mL samples for analysis and formed in 2 mL centrifuge tubes. The samples were sterilised by autoclaving. All analyses were performed with sterile hydrogels fabricated using this method. The samples are shown in Figure 11.

### 4.2. Raman Spectroscopy Analysis

Raman spectroscopy analysis was utilised to determine the correct incorporation of γ-PGA into the WPI hydrogel. The assay utilised an inVia confocal Raman microscope (Renishaw, Gloucestershire, UK). The hydrogels were introduced to the extended spectral analysis utilising a 785 nm laser running at 50% power, with a 5 s exposure time and 10 accumulations. The data were analysed using Origin pro peak analysis software (9.0). Fitting was achieved using Lorentzian fitting and statistical viability was provided through R-squared values and a one-way ANOVA function.

### 4.3. Swelling Analysis

To determine both the effect of the structural behaviour of the hydrogels with the incorporation of γ-PGA and the effect of the neutral pH of the osteo environment on the hydrogels, swelling assays were performed. The method was as follows: WPI-γ-PGA hydrogel samples with a mass of 1 g were introduced to a 5 mL pH 7.4 solution, namely phosphate buffered saline. The initial mass of the hydrogels was taken before the samples were incubated for 1 week. The investigation period was chosen to align with previous WPI investigations. Post incubation, the final mass was taken, and the swelling mass ratio was calculated using the formula below, where the swelling percentage (*S*%) is calculated from the wet mass (*Mw*) and the dry mass (*Md*):

S%=(Mw−Md)/Md×100

### 4.4. Mechanical Testing

The potential of hydrogels to be used in an implant environment requires the load-bearing potential from the hydrogels. WPI hydrogels present poor load-bearing qualities. Therefore, compression analysis was conducted to ascertain if the addition of γ-PGA influenced or increased the load-bearing potential of WPI hydrogels. The analysis was achieved through the use of Instron 3345 (Instron, Norwood, MA, USA). WPI hydrogel samples with γ-PGA concentration percentages of 0%, 2.5%, 5%, and 10% were cut to a height of 10 mm with a diameter of 8 mm. Compression was achieved at a rate of 2 mm/s.

Young’s modulus *Ε* was calculated as

*Ε* = *σ*/*ε* (1)

where *σ* denotes stress and *ε* denotes strain.

Compressive strength *F* was calculated as

*F* = *P*/*A* (2)

where *P* is the load at failure and *A* is the cross-sectional area.

Percentage Strain at break *ε* was calculated as

*ε* = ∆*L*/*L* × 100% (3)

where Δ*L* is the difference between the initial length and the final length and *L* is the initial length.

### 4.5. Cell Culture and Viability

As a model system, an osteoblast precursor cell line MC3T3-E1, derived from mouse calvaria, was utilized for studying cell behaviour in vitro when exposed to various scaffolds. Cells at passages 10 to 14 were cultured in a humidified incubator at 37 °C with 5% CO_2_ in an alpha-MEM medium supplemented with 10% fetal bovine serum (FBS), 2 mM of L-glutamine, 100 μg/mL of penicillin/streptomycin, and 2.5 μg/mL of amphotericin. When they reached confluence, the cells were detached using trypsin/EDTA and then seeded onto the scaffolds. Before seeding the cells, the scaffolds underwent a 10-minute UV irradiation. A suspension of preosteoblastic cells, consisting of 25 × 10 ^ 3 cells per scaffold for the assessment of proliferation and 40 × 10 ^ 3 cells per scaffold for the assessment of differentiation, was introduced into the scaffolds in a 10 μL volume of complete medium. Subsequently, 400 μL of culture medium was added to each scaffold. The culture medium was changed every three days. For differentiation assays, 10 nM of dexamethasone, 10 mM of β-glycerophosphate, and 50 μg/mL of L-ascorbic acid, were added to the primary culture medium.

To assess cell viability, WPI-γ-PGA scaffolds loaded with preosteoblastic cells were subjected to the PrestoBlue™ viability assay [42], which utilizes a resazurin-based indicator. This indicator stains living cells upon uptake, producing a red product that can be photometrically detected. At days 3 and 14 during cell culture, 40 μL of PrestoBlue™ reagent, diluted in alpha-MEM by a factor of 10, was pipetted directly into individual wells, followed by incubation for 60 min at 37 °C. Subsequently, 100 μL of supernatant from each sample was transferred to a 96-well plate, with the help of a Synergy HTX Multi-Mode Microplate Reader (BioTek, Bad Friedrichshall, Germany), and absorbance measurements were performed at 570 and 600 nm. The next step was twofold rinsing of the cell-seeded scaffolds with PBS followed by renewal of their culture media. In all experiments, n = 6. 

### 4.6. Cell Adhesion and Morphology Evaluation using Scanning Electron Microscopy

Scanning electron microscopy (SEM) was used to examine cell attachment and morphology on the scaffolds. Scaffolds, seeded with MC3T3-E1 preosteoblastic cells (25 × 10^3^ cells per sample), were incubated in a 37 °C incubator with 5% CO_2_ for a duration of 7 days. The scaffolds were then rinsed with PBS, fixed for 15 min using a 4% *v*/*v* paraformaldehyde solution, and subsequently dehydrated using ethanol of gradually increasing concentrations (ranging from 30% to 100% *v*/*v*). Afterward, the scaffolds were subjected to drying in a critical point drier (Baltec CPD 030), coated with a 20 nm thick layer of gold using a sputter coater (Baltec SCD 050), and finally observed under a scanning electron microscope at an accelerating voltage of 20 kV (JEOL JSM-6390 LV).

### 4.7. Alkaline Phosphatase (ALP) Activity

To assess ALP activity on days 3 and 7, the scaffolds underwent thorough washing with PBS and subsequent submerging in lysis buffer at pH 10.5 containing 50 mM of Tris-HCl and 0.1% Triton X-100. A total of 350 μL of buffer was used. Subsequently, a series of three freezing and thawing cycles between room temperature and −20 °C were performed. After completion of all three cycles, mixing occurred between a 100 μL suspension of this solution and 100 μL of 2 mg/mL p-nitrophenyl phosphate (pNPP). Prior to mixing, pNPP was subjected to dilution in a buffer which contained 2 mM MgCl_2_ and 50 mM Tris-HCl. The 200 μL mixture resulting from the previous step was subjected to incubation at 37 °C for 1 h. Colour changes were investigated spectrophotometrically at 405 nm. In order to calculate enzymatic activity, the formula [units = nmol p-nitrophenol/min] was used. Normalization to the total cellular protein in the lysates was performed. The Bradford protein concentration assay was used to determine total cellular protein content (AppliChem GmbH, Darmstadt, Germany). For all experiments, n = 6. 

### 4.8. Determination of the Produced Extracellular Collagen

The measurement of total collagen levels secreted by preosteoblastic cells in the culture medium was conducted using the Sirius Red dye assay (Direct red 80, Sigma-Aldrich, St. Louis, MO, USA). Supernatants were collected every 3 days, up to day 21, and 25 µL of each was diluted in deionized water (dd H_2_O) to a total volume of 100 µL, mixed with 1 mL of 0.1% Sirius Red dye, and left to incubate for 30 min at room temperature. Following centrifugation of the samples at 15,000× *g* for 15 min, the resulting pellets were rinsed with 0.1 N HCl to eliminate any unbound dye. Subsequently, the samples were centrifuged at 15,000× *g* for 15 min and dissolved in 500 µL of 0.5 N NaOH. The absorbance was measured using a Synergy HTX plate reader at 530 nm. The absorbance measurements were correlated with a calibration curve of collagen type I concentrations. Experiments were performed for n = 6. 

### 4.9. Measurement of the Concentration of Calcium

The O-cresolphthalein complexone (CPC) method was applied for the quantification of calcium mineralization as a sign of development of the extracellular matrix and osteogenesis [43]. Supernatants were collected every 3 days up to day 21. In this process, 10 μL of culture medium from each sample were mixed with 100 μL of calcium buffer and 100 μL of CPC calcium dye. The absorbance of these solutions was measured at 550 nm after transfer to a 96-well plate. For all experiments, n = 6.

### 4.10. Statistical Analysis

Statistical analysis was performed by conducting an ANOVA *t*-test using GraphPad Prism version 8 software with the aim of determining significant differences between different sample groups and the control at various time points during the experiments. The following p-values were considered to be significant: * *p* < 0.05, ** *p* < 0.01, *** *p* < 0.001, and **** *p* < 0.0001, while “ns” represented an insignificant difference relative to the WPI control scaffold at the corresponding time point.

## Figures and Tables

**Table 1:** Suggested underlying interactions in the γ-PGA–WPI Raman spectroscopy results.

WPI	WPI/2.5	WPI/5	WPI/10%	Interaction	Reference
128	129	130	131	Lattice rocking vibrations	[27]
162	159	159	172	CO_2_ torsion, lattice rocking vibrations	[27]
217	210	214	269	L-glutamic acid skeleton vibrations	[27]
			758–852	CH_2_ rocking vibrations, COOH deformation vibrations	[28]
1024	1007	1004	986	CC stretching vibrations, C–C–N stretching vibrations	[27,29]
	1155		1121	NH_3+_ rocking vibrations, C–O stretching vibrations, CH_2_ twisting vibrations	[27,28]
1243	1242		1249	C–O stretching vibrations, CH_3_ wagging vibrations, COH in plane bending vibrations, CH_3_COOH (H-bonded)	[28]
1328	1331	1321	1331	COH in plane bending vibrations, CH_3_ wagging vibrations, CH in plane bending vibrations, COO– symmetric stretching vibrations	[28,29]
1451	1451	1451	1494	CH_3_ antisymmetric in plane bending vibrations, COO– symmetric stretching vibrations, COH in plane bending vibrations	[28,29]
1542	1552		1542	COO– anti-symmetric stretching vibrations	[28]
1665	1666	1666	1669/1763	C=O stretching vibrations	[27]
2928	2928	2928	2928	CH_2_ stretching vibrations	[27]

**Table 2:** Composition percentages of WPI-γ-PGA hydrogels.

Sample	% WPI	% γ-PGA
WPI	40	0
WPI/2.5	40	2.5
WPI/5	40	5
WPI/10	40	10

## Data Availability

Data are contained within the article.
